# Autophagy and the Cell Cycle: A Complex Landscape

**DOI:** 10.3389/fonc.2017.00051

**Published:** 2017-03-31

**Authors:** Søs Grønbæk Mathiassen, Daniela De Zio, Francesco Cecconi

**Affiliations:** ^1^Cell Stress and Survival Unit, Danish Cancer Society Research Center, Copenhagen, Denmark; ^2^Department of Biology, University of Rome Tor Vergata, Rome, Italy; ^3^Department of Pediatric Hematology and Oncology, Istituto di Ricovero e Cura a Carattere Scientifico Bambino Gesù Children’s Hospital, Rome, Italy

**Keywords:** autophagy, cancer, cell cycle, cell stress, senescence, mitosis, cytokinesis, p53

## Abstract

Autophagy is a self-degradation pathway, in which cytoplasmic material is sequestered in double-membrane vesicles and delivered to the lysosome for degradation. Under basal conditions, autophagy plays a homeostatic function. However, in response to various stresses, the pathway can be further induced to mediate cytoprotection. Defective autophagy has been linked to a number of human pathologies, including neoplastic transformation, even though autophagy can also sustain the growth of tumor cells in certain contexts. In recent years, a considerable correlation has emerged between autophagy induction and stress-related cell-cycle responses, as well as unexpected roles for autophagy factors and selective autophagic degradation in the process of cell division. These advances have obvious implications for our understanding of the intricate relationship between autophagy and cancer. In this review, we will discuss our current knowledge of the reciprocal regulation connecting the autophagy pathway and cell-cycle progression. Furthermore, key findings involving nonautophagic functions for autophagy-related factors in cell-cycle regulation will be addressed.

## Introduction

Macroautophagy (herein referred to as autophagy) is a highly conserved catabolic pathway that mediates the sequestration and delivery of cytoplasmic material to the lysosome for degradation. This is achieved by the formation and expansion of an isolation membrane (or phagophore) that fuses to engulf cytoplasmic constituents in a double-membrane autophagic vacuole (the autophagosome). The autophagosome finally undergoes fusion with lysosomes whereby the enclosed cargo is degraded and subsequently released and recycled to support cellular metabolism. In physiological conditions, autophagy proceeds at a basal level to ensure the turnover of superfluous or damaged components, including organelles and long-lived proteins, to maintain cellular homeostasis. Moreover, the autophagic flux can be upregulated in response to a wide range of stresses, such as nutrient deprivation, reactive oxygen species, DNA damage, protein aggregates, damaged organelles, or intracellular pathogens, whereby it functions as an adaptive cytoprotective response (1, 2).

**Figure 1:**
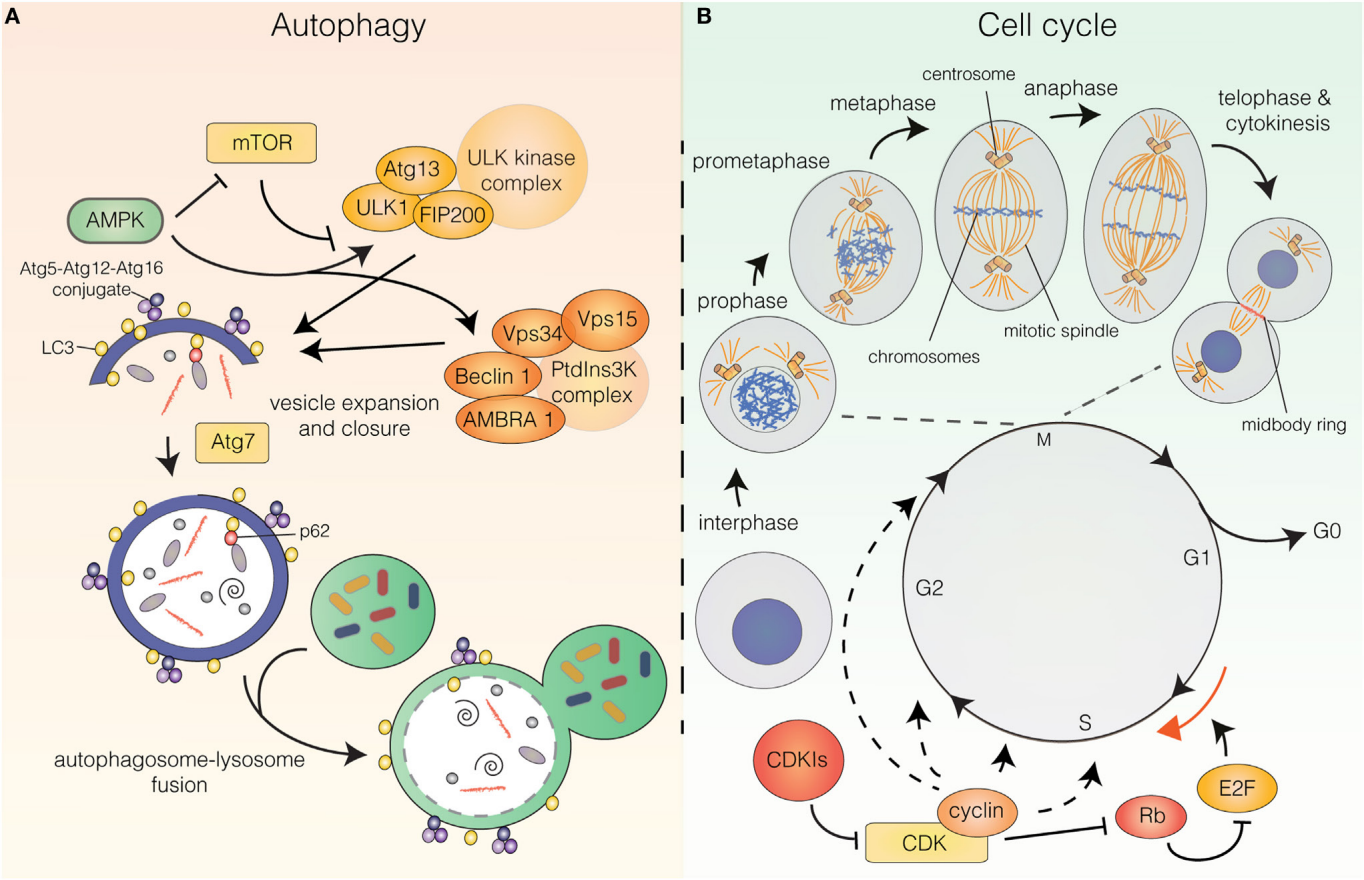
**(A)** The autophagy pathway. Autophagy induction is controlled upstream by energy sensors, mammalian target of rapamycin (mTOR), and AMP-activated protein kinase (AMPK). mTOR shuts off autophagy in the presence of abundant nutrients, while AMPK is activated upon energy stress. AMPK induces autophagy by inhibiting mTOR and stimulating upstream autophagy factors of the unc-51-like autophagy-activating kinase (ULK) and class III phosphatidylinositol 3-kinase (PtdIns3K) complexes. Vesicle expansion requires the autophagy-related gene 8 (Atg8)/light chain 3 (LC3) and autophagy-related gene 12 (Atg12) ubiquitin-like conjugation systems. Autophagy receptors (e.g., p62) can mediate selective recruitment of cargo to the inner vesicle membrane. Following vesicle closure, the autophagosome fuses with the lysosome whereby the engulfed material is degraded. **(B)** The cell cycle. The cell cycle can be divided into G0, G1, S, G2 (interphase), and M-phase (mitosis and cytokinesis). Mitosis can be subdivided into prophase (DNA condensation is initiated), prometaphase (the mitotic spindle starts to form and the nuclear envelope has been dissolved), metaphase (the chromosomes are aligned at the metaphase plate), anaphase (separation of the sisterchromatids) and telophase (DNA decondenses, the nuclear envelope reforms, the contractile ring starts forming) and is followed by cytokinesis (physical separation of the daughter cells). Cell-cycle progression is governed by cyclin-dependent kinase (CDK) holoenzymes. CDK activity can be inhibited by cyclin-dependent kinase inhibitors. For G1/S transition cyclin-CDKs phosphorylate retinoblastoma protein (Rb), which releases E2 factor (E2F) transcription factors from inhibitory binding, leading them to induce transcription of targets for G1/S transition.

The molecular pathway that orchestrates the initiation and execution of autophagy has been comprehensively reviewed elsewhere (3–5). In short, the initiation phase of autophagy is governed by two main complexes: the unc-51-like autophagy-activating kinase (ULK) complex and the class III phosphatidylinositol 3-kinase (PtdIns3K) complex (Figure 1A). The PtdIns3K complex produces phosphatidylinositol 3-phosphate (PtdIns3P) for recruitment of additional autophagy factors to the phagophore and is partially comprised of the key autophagy regulators vacuolar protein sorting 34 (Vps34), Beclin 1, vacuolar protein sorting 15 (Vps15), and activating molecule in Beclin 1-regulated autophagy (AMBRA1). Downstream of these complexes are two ubiquitin-like conjugation systems that mediate vesicle expansion [the autophagy-related gene 8 (Atg8) and autophagy-related gene 12 (Atg12) systems]. Both systems require the E1-like protein autophagy-related gene 7 (Atg7) for activation of the ubiquitin-like proteins Atg8 [light chain 3 (LC3) in mammals] and Atg12. In the Atg8 system, Atg8/LC3 undergoes proteolytic processing and covalent attachment to the lipid phosphatidylethanolamine (in mammalian cells, the precursor form is termed LC3-I and the lipidated form LC3-II), by which it becomes associated with the phagophore membrane. Consequently, autophagy can be detected biochemically (by assessing the generation of LC3-II) or microscopically (by observing the formation of LC3 puncta, representative of LC3 redistribution to the developing autophagosomes). Apart from these systems, the pathway includes the transmembrane protein autophagy-related gene 9 (Atg9), as well as factors involved in autophagosome–lysosome fusion [e.g., lysosomal-associated membrane protein 2 (LAMP2)], vacuolar permeases mediating the efflux of amino acids from the lysosome, and lysosomal enzymes required for cargo degradation (3–7). Furthermore, while originally considered a largely unspecific process, recent years have revealed the existence of selective autophagy pathways, in which specific cargoes can be targeted to the emerging autophagosomes for engulfment and degradation. Cargoes destined for selective autophagy are often ubiquitinated and recognized by autophagy receptors [i.e., p62/sequestosome 1, neighbor of BRCA1 gene (NBR1), nuclear dot protein 52 kDa (NDP52), optineurin, or C-Cbl] that contain ubiquitin-binding domains as well as LC3-interacting region (LIR) motifs for recruitment to the inner phagophore membrane (8, 9) (Figure 1A).

Autophagy induction is controlled upstream by energy-sensing proteins, a key regulator being the mammalian target of rapamycin (mTOR), which provides the major inhibitory signal that shuts off autophagy in the presence of abundant nutrients. A key inhibitor of mTOR AMP-activated protein kinase (AMPK) is activated upon energy stress that increases the AMP/ATP ratio. Once activated, AMPK downregulates ATP-consuming (anabolic) pathways and upregulates ATP-generating (catabolic) pathways, such as autophagy, to maintain cellular energy homeostasis. Besides inhibiting the catalytic activity of mTOR, AMPK also directly stimulates autophagy by phosphorylating upstream autophagy factors [e.g., unc-51-like autophagy-activating kinase 1 (ULK1) and Beclin 1] (1, 3, 4) (Figure 1A).

In recent years, the notion that autophagy may represent a bona fide tumor suppressor pathway has obtained increasing support. Autophagy-deficient animal models are often prone to tumor formation (10–16) and autophagy deficiency is associated with increased DNA damage and chromosomal instability (CIN) (17). Thus, autophagy is thought to constitute a barrier against malignant transformation by preserving intracellular homeostasis, even though the exact mechanism of autophagy-mediated oncosuppression is not well-understood. Autophagy can conversely sustain the survival and proliferation of neoplastic cells exposed to intracellular and environmental stresses, such as hypoxia and chemotherapy, and thereby supports tumor growth and progression. Hence, depending on the context, autophagy can act either as a tumor-suppressive or a tumor-promoting pathway (2, 18, 19).

As many signaling pathways exhibit opposing effects on autophagy and cell-cycle progression (20), these are often considered mutually exclusive processes. Accumulating evidence suggests that this opposing regulation may be coordinated and that an interplay between the two processes exists. This is exemplified by the scaffold protein AMBRA1, a pro-autophagic protein that is also able to negatively regulate the oncogene c-Myc (10). AMBRA1 interacts with the catalytic subunit of the protein phosphatase 2 A (PP2A) and facilitates PP2A-mediated dephosphorylation and subsequent proteasomal degradation of c-Myc, thus resulting in inhibition of proliferation and in tumor suppression (10). Both the role of AMBRA1 in promoting c-Myc degradation, as well as in AMBRA1-dependent autophagy, is controlled upstream by mTOR (10, 21), which argues for a coordinated regulation of autophagy and cell-cycle progression.

In the present review, we will focus on various aspects of the reciprocal regulation connecting autophagy and the cell cycle. Cell-cycle progression is governed by cyclin-dependent kinases (CDKs). CDK activity is coordinated by binding of their essential regulatory subunits, cyclins, which are synthesized and degraded at specified times during the cell cycle to coordinate timely CDK activation and drive cell-cycle progression (Figure 1B). The decision to enter or exit the cell cycle depends on the nutrient and mitogen availability and is also affected by stress-stimuli that may block the cell cycle transiently or irreversibly. Once committed to cell-cycle progression, the cell undergoes a series of regulated events (i.e., cell growth, DNA replication, and quality control checkpoints) culminating in the highly orchestrated process of cell division. Dysregulation of proteins controlling the frequency and fidelity of proliferation is inextricably linked to neoplastic transformation (22–24).

Herein, we will address the activation of autophagy during normal and abnormal cell-cycle progression as well as the coordinated induction of autophagy and cell-cycle responses following exposure to various stresses. Finally, the involvement of autophagy and autophagy-related proteins in the regulation of cell division will be discussed.

## Autophagy Status During Cell-Cycle Progression

Only few studies have focused on a putative correlation between autophagy flux and cell-cycle progression. The cell cycle can be divided into five major phases: G_0_, G_1_, S, G_2_, and M-phase (Figure 1B). G_0_, G_1_, S, and G_2_ are collectively referred to as interphase, while M-phase is comprised of mitosis and cytokinesis, the processes by which the duplicated genome and other cellular constituents are distributed to the two daughter cells and the subsequent separation of these. Mitosis is traditionally subdivided into five phases: prophase (DNA condensation is initiated), prometaphase (the mitotic spindle starts to form and the nuclear envelope is dissolved), metaphase (the chromosomes are aligned at the metaphase plate), anaphase (separation of the sister chromatids to separate chromosomes) and telophase (DNA decondenses, the nuclear envelope is reformed and the contractile ring at the intercellular bridge between the two nuclei starts forming). This is followed by cytokinesis, in which the two daughter cells are physically separated (25) (Figure 1B).

### Autophagy and Interphase

The question of differential regulation of autophagy during cell-cycle progression was initially addressed by Tasdemir et al., prompted by their observation that autophagy-inducing treatment of unsynchronized cell populations only induced green fluorescence protein (GFP)–LC3 aggregation in approximately 50% of cells (26). To understand if autophagy preferentially occurs in certain cell-cycle phases, immunocytochemical approaches were employed to monitor cytoplasmic GFP–LC3 aggregation in connection with cell-cycle progression (26). Using a panel of autophagy activators, including the BH3 mimetic ABT737, lithium, rapamycin, tunicamycin, or starvation, autophagy induction was observed to preferentially occur in the G1 and S phases of the cell cycle (26). More recently, Kaminskyy et al. developed another strategy to monitor autophagosome accumulation by extracting membrane-unbound LC3-I from cells, followed by flow cytometric detection of the remaining autophagosomal membrane-associated fraction of LC3-II. This was combined with propidium iodide staining for detection of cell-cycle status (27). By using this approach, basal autophagy was detected in G1, S, and G2/M phases. Furthermore, autophagy induction by starvation or rapamycin treatment resulted in LC3-II accumulation in all stages (27), suggesting the absence of cell-cycle-dependent autophagy regulation. The contradictory findings may be the result of the variant experimental approaches. Thus, further studies are required to determine if autophagy activation is preferentially linked to specific cell-cycle phases.

### Autophagy and Mitosis

As the above studies do not allow discrimination between G2 and M phase, this leaves the question of autophagy status during mitosis. Two elegant studies have reported a striking decrease in autophagic activity during mitosis (28, 29). By means of electron microscopy and stereology to quantify the presence of autophagic vacuoles in mitotic cells, Eskelinen et al. found a strong reduction in autophagosomal content in both (pro)metaphase and anaphase cells (28). Furuya et al. expanded on these findings revealing that mitotic autophagy inhibition depends on cyclin-dependent kinase 1 (CDK1)-mediated phosphorylation of Vps34 on Thr159 during mitosis (29). This phosphorylation event negatively regulates the interaction between Vps34 and Beclin 1, thereby inhibiting PtdIns3K activity, PtdIns3P production, and autophagy induction (29). Of note, during mitosis, cells undergo extensive structural rearrangements and the inhibition of autophagy has been speculated to function as a protective mechanism to prevent unintended loss of organelles and chromosomes. Indeed, break down of the nuclear envelope during mitosis leaves the condensed chromosomes potentially vulnerable to the cytoplasmic autophagy machinery. Accordingly, Eskelinen et al. observed that re-appearance of autophagosomes occurred in telophase/G1 after formation of the new nuclear envelopes (28). Furthermore, autophagosomal engulfment of mitotic chromosomes was reported in mitotic cells undergoing programmed cell death (30), suggesting that autophagy inhibition may, indeed, protect the condensed genome from accidental autophagic engulfment. Moreover, during cell division, mitochondria and the Golgi apparatus become fragmented to facilitate their distribution between the two daughter cells (31, 32). While elongated mitochondria are spared from autophagic degradation (33, 34), the smaller size of fragmented mitochondria facilitates their uptake by autophagosomes (35, 36). Mitotic fragmentation of mitochondria is mediated by CDK1-dependent phosphorylation and activation of the dynamin-like protein (Drp1), involved in mitochondrial fission (37). Interestingly, cells arrested in mitosis by abrogated Cyclin B1 degradation, exhibit a gradual decline in mitochondrial mass due to ongoing mitophagic degradation (38). Prevention of mitophagy by depletion of Drp1 or key autophagy proteins delayed cell death by mitotic arrest; thus, mitophagy may facilitate mitotic cell death during prolonged mitotic block (38). The resistance to mitotic cell death acquired upon Drp1 knock-down supports the speculated vulnerability of fragmented mitotic mitochondria to autophagic degradation. Ongoing mitophagy during mitotic arrest may simply represent leaky degradation from incompletely blocked autophagy, which is functionally relevant during prolonged mitotic arrest but likely negligible during normal mitotic progression. However, this mechanism may also participate in pushing cells with mitotic abnormalities toward cell death.

In accordance with the reported ongoing mitophagy in arrested mitotic cells (38), LC3 puncta have been observed in mitotic cells, although at a significantly decreased level compared to interphase cells (28, 29, 39, 40). While these may also represent inefficient autophagy inhibition, Loukil et al. observed LC3, p62, and lysosomal markers colocalizing with Cyclin A2 foci during mitosis and found that autophagy partially contributes to mediating mitotic Cyclin A2 degradation (40). Thus, an intriguing although highly controversial theory is the existence of distinct sites of active autophagy during cell division. Treatment with autophagy inducers or lysosomal inhibitors has been shown to result in accumulation of LC3 puncta in mitotic cells, which was suggested as an indication of active autophagy flux in mitosis (39, 41). The short duration of mitosis, however, poses technical challenges in employing these treatments, as it is difficult to rule out autophagosome accumulation from interphase. Live-cell imaging using GFP–LC3 cell lines or preferably cell lines carrying endogenously tagged autophagy proteins may help in determinining the degree of autophagy inhibition as well as the potential presence of active autophagic compartments in mitosis.

## Interplay Between Autophagy and Cell-Cycle Arrest

In response to unfavorable or stressful conditions, cells are able to arrest the cell cycle transiently or irreversibly. This ability helps regulate proliferation during development and differentiation, and prevents the expansion of potentially harmful cell populations (23, 42). Autophagy, like cell cycle arrest, is induced in response to a variety of stress conditions, where it plays a pivotal role in preserving cellular viability (2). While the correlative induction of autophagy and cell-cycle arrest has been extensively documented, the molecular mechanisms linking them together are still debated and largely unknown.

### Autophagy Regulation by Cyclin-Dependent Kinase Inhibitors (CDKIs) and Retinoblastoma Protein (Rb)/E2 Factor (E2F) Activity

Cell-cycle arrest often relies on the action of various cell-cycle inhibitors. An important class of those are CDKIs that inhibit CDK activity by direct interaction with CDKs or cyclin-CDK holoenzymes (43) (Figure 1B). CDKIs can be categorized into two main families: the inhibitors of CDK4 (INK4) family consisting of p15^INK4B^, p16^INK4A^, p18^INK4C^, and p19^INK4D^; and the Cip/Kip family composed of p21^Cip1^, p27^Kip1^, and p57^Kip2^ (23, 42). In spite of their similar modes of action, CDKIs are speculated to have functionally distinct roles and appear to be activated by different stimuli (42). Thus, while p21 is most strongly linked to stress and DNA damage signaling, downstream of p53-mediated pathways, p27 is more often associated with cell-cycle arrest in response to low nutrient and mitogen conditions (42). CDKIs were originally strictly linked to proliferation control, but they are now demonstrated to have a wide range of alternative functions in processes including transcription, apoptosis, migration (42), as well as autophagy induction (44–46). Cell-cycle arrest can also occur by repression of E2F transcription factors that mediate transcriptional induction of a plethora of targets, including cyclins and replication regulators required for G1/S transition and cell-cycle progression (47, 48) (Figure 1B). E2F activity is controlled by binding of the Rb protein or other Rb family members (49). Upon mitogenic stimuli, Rb is gradually phosphorylated by cyclin-CDK complexes whereby E2F is released to induce transcription of its target genes, pushing cells to pass the G1/S boundary (47, 49). CDKIs, through their ability to inhibit CDKs, are also important indirect promoters of Rb/E2F interactions (48) (Figure 1B).

A number of CDKIs, including p16, p21, and p27 have been reported to induce autophagy (44–46), suggesting the existence of coordinated stress responses linking autophagy induction and cell-cycle arrest. Liang et al. showed that in response to starvation, p27 is activated by the liver kinase B1 (LKB1)–AMPK nutrient-sensing pathway through phosphorylation of Thr198, thus resulting in p27 stabilization (46). Interestingly, p27 was required for efficient starvation-induced autophagy in murine embryonic fibroblast and protected from cell death resulting from metabolic stress (46), indicating a critical role for p27 in autophagy activation under starvation conditions. The increased stability of p-p27^Thr198^ implies a function for the LKB1–AMPK pathway in mediating p27-dependent cell-cycle arrest. Accordingly, a nonphosphorylatable p27^T198A^ mutant was less efficient than wild type p27 or a phospho-mimicking p27^T198D^ mutant at inhibiting colony formation (46). This is in line with previous reports arguing for a central role for p27 in starvation-induced cell-cycle arrest (50, 51). p27 is upregulated in response to serum starvation (50) and its depletion allows serum-starved cells to evade cell-cycle arrest and continue proliferation (50, 51). Thus, p27 may be a key effector of the cellular response to metabolic stress, functioning downstream of the LKB1–AMPK axis to mediate both cell-cycle arrest and autophagy induction. Accordingly, p27 is degraded by caspases during growth-factor deprivation-induced apoptosis (52).

The mechanism by which p27 mediates autophagy induction and the relevance of its CDK inhibitory function in this context is, however, not clear. Nonetheless, it has been reported that the cyclin-binding region of p27 is required for autophagy induction (46, 53) and that depletion of CDK2 and CDK4 partially reproduces p27-induced effects on autophagy and apoptosis (46). In this context, indirect activation of Rb by p27 could be a contributing factor, as this has been reported for p16 (45). Overexpression of p16 is able to induce autophagy in an Rb-dependent manner through promoting Rb/E2F interaction (45), which suggests negative regulation of autophagy by E2Fs. This supports a model in which p16-mediated CDK inhibition facilitates Rb/E2F interaction and consequent E2F inhibition, resulting in activation of autophagy through an unspecified mechanism. However, while autophagy induction by p16 appears to largely depend on Rb/E2F regulation, p27-induced autophagy was only mildly affected by Rb status (45), suggesting varying mechanisms of autophagy activation between CDKIs. Intriguingly, in budding yeast, the CDK Pho85 is able to both induce or inhibit autophagy, depending on its associated cyclin partner (54).

The literature linking Rb/E2F and autophagy is complex, as positive regulation of autophagy by E2Fs has also been reported. Using an inducible E2F activation system, Polager et al. demonstrated that several autophagy genes such as LC3, ULK1, and DRAM were direct targets of E2F transcription factors (55). Moreover, E2Fs were shown to bind the promoter region of Beclin 1 (56), even though the functional significance of this binding remains to be demonstrated. E2F downstream targets such as smARF or the hypoxia-inducible B-cell lymphoma 2 (Bcl-2) family member BCL2 interacting protein 3 (BNIP3) have also been shown to induce autophagy (44, 57, 58). BNIP3 was demonstrated to be required for efficient hypoxia-induced autophagy activation (58) and E2F1 to be required for efficient DNA-damage-induced autophagy (55). This evidence indicates a potential role for E2Fs in mediating autophagy during acute stress responses, rather than during normal cell-cycle progression. E2F-mediated autophagy induction may therefore depend on the context and stimuli. Furthermore, as the E2F family comprises eight family members that can both transactivate and repress gene expression (47), E2F contribution to autophagy regulation likely depends on the involved E2F factor.

### Autophagy and p53

**Figure 2:**
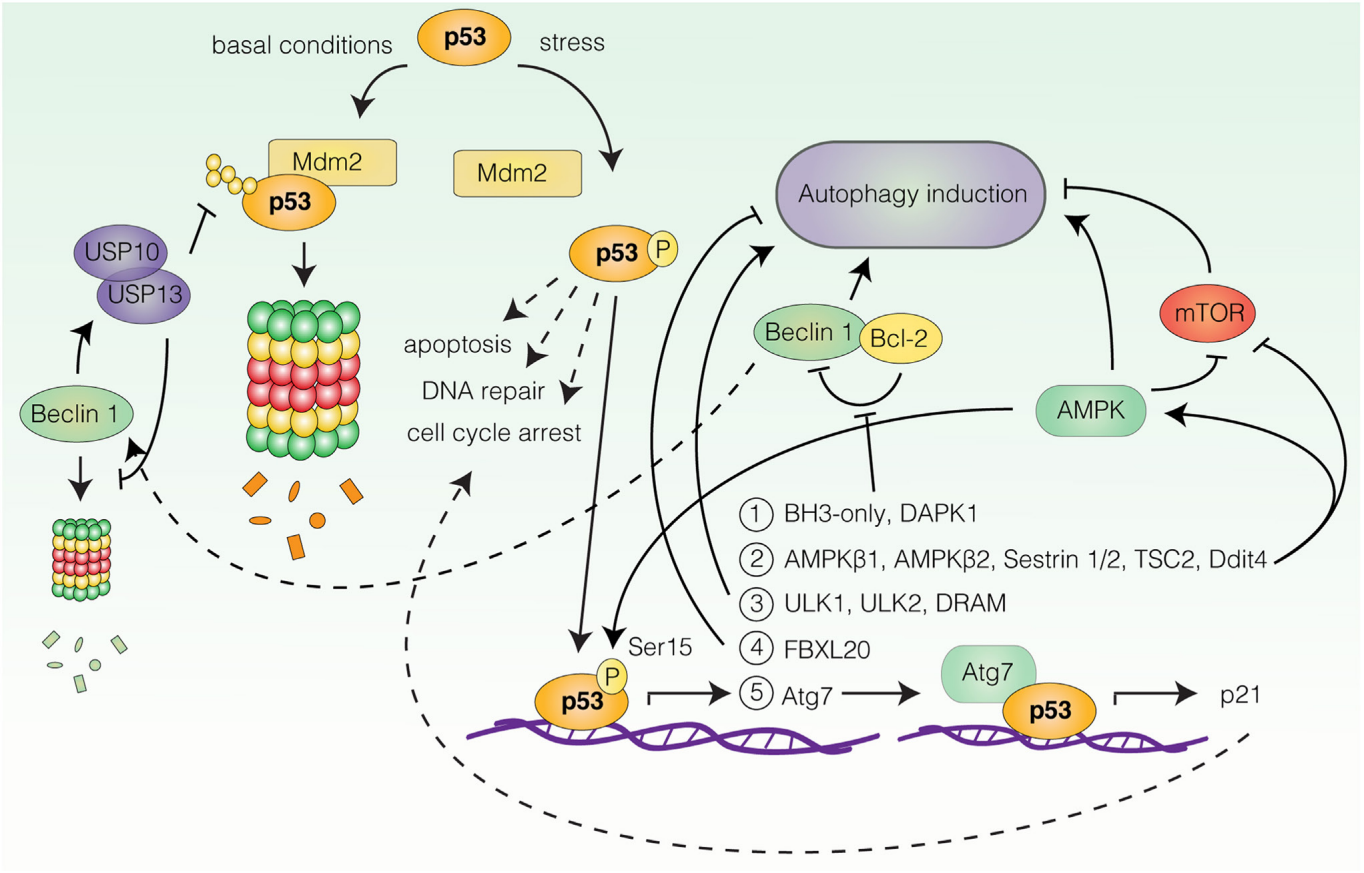
**Transcriptional regulation of autophagy by p53**. Under basal conditions p53 is degraded by mouse double minute 2 homolog (Mdm2)-mediated proteasomal degradation. In response to stress, p53 undergoes post-translational modifications leading to its stabilization and activation. Upon activation, p53 can induce transcription of autophagy-related genes (only a selection is represented here). Group 1: BH3-only proteins and death-associated protein kinase 1 (DAPK1), all stimulate autophagy by favoring Beclin 1 displacement from B-cell lymphoma 2 (Bcl-2) and B-cell lymphoma extra large (Bcl-X_L_). Beclin 1 can contribute to p53 stabilization by stabilizing the deubiquitinating enzymes ubiquitin-specific peptidase 10/13 (USP10/13). Group 2: AMP-activated protein kinase (AMPK) subunits β1 and β2, AMPK activators Sestrin 1/2, negative regulators of mammalian target of rapamycin (mTOR), tuberous sclerosis 2 (TSC2), and DNA damage-inducible transcript 4 (Ddit4), all promote autophagy induction. AMPK can in turn phosphorylate and activate p53. Group 3: unc-51-like autophagy-activating kinase 1 (ULK1), unc-51-like autophagy-activating kinase 2 (ULK2), and damage-regulated autophagy modulator. Target 4: F-box/LRR-repeat protein 20 (FBXL20) negatively regulates autophagy by promoting vacuolar protein sorting 34 (Vps34) degradation. Target 5: Key autophagy protein autophagy-related gene 7 (Atg7) cooperates with p53 for p21 induction.

The most well-documented connection between autophagy and stress-induced cell-cycle responses is likely the link between p53 and autophagy regulation. p53 is one of the most extensively characterized tumor suppressor proteins and a central coordinator of the cellular response to acute stress (59, 60). Under basal conditions, p53 levels are strictly controlled by mouse double minute 2 homolog (Mdm2)-mediated ubiquitination and proteasomal degradation, while in response to a wide range of stresses (e.g., DNA damage, oncogene expression or nutrient deprivation), p53 undergoes rapid post-translational modifications that allow for its stabilization and activation (59) (Figure 2). Upon activation, p53 orchestrates the induction of appropriate cellular responses, be it apoptosis, cell-cycle arrest, DNA repair, metabolic adaptation, or autophagy, with the purpose of limiting the expansion of damaged and potentially harmful cells (59, 60) (Figure 2). The shared involvement of p53 and autophagy in stress-related processes, as well as their relevance for neoplastic transformation has motivated great efforts to understand the role of autophagy ablation in the context of p53-deficient and -proficient animal models of human cancers, reviewed in Ref. (61). In this article, we will focus our attention on the molecular mechanisms linking p53 to autophagy regulation.

#### Autophagy Modulation by Nuclear p53

A number of reports have demonstrated autophagy induction by p53 (18, 62, 63). The ability of p53 to stimulate autophagy appears to rely on its function as a stress-induced transcription factor, as p53 can transactivate a wide range of autophagy-related genes (18, 62, 63) (Figure 2). Activation of some of these genes converges on activation of AMPK and inhibition of mTOR. These include genes encoding the AMPKβ1 and β2 subunits (64), the AMPK activators Sestrin 1 and Sestrin 2 (65, 66), as well as negative regulators of mTORC1, tuberous sclerosis 2 (TSC2), phosphatase and tensin homolog (PTEN), and DNA damage-inducible transcript 4 (Ddit4) (64, 67, 68). Accordingly, Feng et al. reported that p53-induced autophagy following DNA damage relied on AMPK-mediated inhibition of mTOR (69). Other p53 responsive genes include ULK1 and unc-51-like autophagy-activating kinase 2 (ULK2) (70), genes encoding various BH3-only proteins and death-associated protein kinase 1 (DAPK1), all of which stimulate autophagy by favoring the displacement of Beclin 1 from inhibitory interactions with Bcl-2 and Bcl-X_L_ (71–74), as well as the gene coding for DRAM (75), a highly conserved lysosomal protein, which was also suggested to be required for p53-dependent autophagy induction in response to DNA damage (75). Furthermore, Kenzelmann Broz et al. utilized a high-throughput approach to uncover novel p53 transcriptional targets in response to DNA damage (67). This approach identified extensive transactivation of the autophagy network, encompassing both upstream autophagy regulators, members of the autophagy core machinery, and lysosomal proteins by all three p53 family members; p53, p63, and p73 (67). Interestingly, one of the identified targets, Atg7 (67), has been reported to bind the promoter of p21, collaborating with p53 for efficient p21 upregulation in a nonautophagy-dependent manner (76) (Figure 2). Thus, p53-dependent upregulation of Atg7 may function as an effector mechanism boosting the p53 response through p21 production. Similarly, AMPK can activate p53 upon glucose deprivation by phosphorylation of Ser15, which is required for AMPK-mediated cell-cycle arrest in this context (77) (Figure 2). Surprisingly, Beclin 1 can also contribute to p53 stabilization by promoting the stabilization of deubiquitinating enzymes ubiquitin-specific peptidase 10/13 (USP10/13) (78), which counteract the Mdm2-mediated degradation of p53 (78, 79), as well as degradation of Beclin 1 itself (78) (Figure 2). Thus, autophagy and p53 pathways may potentiate and sustain each other in establishing efficient stress-related cell-cycle programs.

Interestingly, activated p53 is also able to decrease autophagy, as the p53-responsive gene F-box/LRR-repeat protein 20 (FBXL20) is able to mediate the degradation of Vps34 following DNA damage, resulting in autophagy inhibition (80). In which context p53 activation results in autophagy stimulation and inhibition, respectively, is not understood. Furthermore, the effect of p53-induced autophagy is not clear, but in several contexts autophagy surprisingly appears to function as an effector of p53-mediated cell death rather than as a survival mechanism (67, 70, 75).

#### Autophagy Inhibition by Cytosolic p53

Contrasting the proautophagic transcriptional activity of nuclear p53, the cytoplasmic pool of p53 has been demonstrated to suppress autophagy (81). Knockout, depletion, or pharmacological inhibition of p53 in human, mouse as well as nematode cells, can induce autophagy in a manner appearing to depend on the AMPK/mTOR pathway (81). Correspondingly, p53 restricted to the cytosol but not nucleus-restricted p53 inhibited autophagy, a regulation that also persisted in enucleated cells (81). Accordingly, suppression of autophagy by p53 correlated with its nuclear-to-cytosolic distribution in a panel of cancer-associated p53 mutants (82). Surprisingly, several distinct proautophagic stimuli, including nutrient deprivation and mTOR inhibition by rapamycin were found to induce Mdm2-dependent proteasomal degradation of p53. Inhibition of proteasomal activity, Mdm2 depletion, or pharmacological inhibition of Mdm2 reduced autophagy induction in response to these stimuli (81), suggesting the requirement of p53 degradation for efficient autophagy activation. The molecular mechanism underlying this p53-mediated autophagy suppression is not understood, but has been suggested to involve negative regulation of the upstream autophagy factor RB1 inducible coiled-coil 1/FAK family kinase-interacting protein of 200 kDa (RB1CC1/FIP200) through a physical interaction with p53 (83). How the contradictory regimes of cytoplasmic versus nuclear p53-mediated autophagy regulation can be reconciled remains to be determined.

### Autophagy and Senescence

While several lines of evidence suggest coordinated induction of autophagy and cell-cycle arrest pathways, another issue remains the involvement of autophagy in the execution of cell-cycle exit programs, in particular, *senescence*. The terms *quiescence* and *senescence* are often used interchangeably to describe cell-cycle arrest, although they refer to distinct cell states (84). *Quiescence* represents a reversible cell-cycle arrest often caused by lack of nutrients and/or mitogens and growth factors, while *senescence* is an irreversible state of cell-cycle arrest that is more often induced in abnormal (potentially cancerous), DNA-damaged, or aging cells as a stress response (84–86). While it is clear that autophagy and senescence are often parallel processes, the question of their interdependence is a subject of much debate. It is beyond the scope of the present review to comprehensively recapitulate the literature involving this topic, and for more on this subject, we refer to Ref. (84, 87, 88). In this article, we will focus our attention on key findings and recent publications that offer mechanistic insight to the relationship between autophagy and senescence.

#### Autophagy and Senescence Transition

In recent years, a number of studies have argued for a more direct link between autophagy and senescence that goes beyond their correlative induction, by showing that inhibition of autophagy delays senescence transition (89–93). Young et al. employed models of oncogene-induced and DNA damage-induced senescence to study autophagy activation during senescence transition (93). In the applied model of oncogene-induced senescence (OIS), an initial “mitotic phase” of proliferative burst occurs around day 1. This is followed by a “transition phase,” preceding the “senescence phase,” which is achieved after 5–6 days. Autophagy was induced specifically in the senescence transition phase in a manner that correlated with inhibition of mTOR activity. Importantly, Young et al. observed that depletion of the autophagy proteins autophagy-related gene 5 (Atg5) or Atg7 resulted in delayed senescence transition (93), thus indicating that autophagy contributes to the establishment of senescence. Similar results were obtained in a system of therapy-induced senescence, in which pharmacological or genetic inhibition of autophagy delayed senescence acquisition in response to treatment with the chemotherapeutic drugs adriamycin or camptothecin (90). In accordance with these findings, a recent study expands on a putative mechanism of autophagy-mediated senescence transition, as Dou et al. found that autophagy facilitates OIS by degrading the nuclear lamina constituent, Lamin B1, and associated heterochromatin domains called lamin-associated domains (LADs) (89). Degradation was a result of nuclear blebbing of Lamin B1 regions and a direct interaction between Lamin B1 and LC3, and preferentially occurred in response to oncogenic transformation, oxidative stress, and DNA damage, but not starvation (89), indicating that the degradation event is specific to a subset of stresses. Senescence was delayed upon expression of Lamin B1 mutants unable to bind LC3 and undergo autophagic degradation (89). Thus, autophagic Lamin B1 degradation may be of key importance during senescence transition. Interestingly, senescent cells have previously been shown to exhibit a gradual decline in histone mass that was dependent on lysosomal activity (94). Whether the degradation of Lamin B1-associated chromatin is of relevance for senescence transition is an interesting point for further investigation. Furthermore, autophagy was found to mediate the selective degradation of Δ133p53α (95), a p53 isoform suppressing the action of full-length p53 (96, 97), for induction of replicative senescence but not OIS (95, 97). Interestingly, overexpression of autophagy proteins is, in some cases, sufficient to stimulate coordinated induction of autophagy and premature senescence (93, 98). Nonetheless, as autophagy inhibition, in most cases, delays rather than fully abrogates senescence, it has been argued that autophagy is not required for senescence transition, but may function in potentiating and accelerating the response (87).

It should also be noted that active mTOR is demonstrated to have a key role in favoring senescence over quiescence and may even be a requirement for senescence transition and/or maintenance in many contexts (99–104). In fact, the main characteristics of senescent cells include hyperactive features such as cellular hypertrophy and the senescence-associated secretion phenotype, which require high metabolic activity (84, 104), and have been speculated to be in part the result of uncoupling proliferation and mTOR activity (85, 105). It should therefore follow that an intrinsic feature of senescent cells would be decreased autophagic activity, as has indeed been demonstrated in some reports (106). However, Narita et al. intriguingly described the formation of a compartment termed the mTOR-autophagy spatial coupling compartment (TASCC) upon OIS, in which mTOR and lysosomes are enriched in the vicinity of the rough endoplasmic reticulum–Golgi apparatus (107). The TASCC was speculated to shield mTOR from the upstream autophagy factors it usually inhibits (4, 107), allowing for concurrence of protein synthesis and degradation, while strategically situating mTOR and lysosomes in a favorable context for mTOR activation on the lysosomal surface (107, 108). In addition, an increasing number of reports have identified pathways and molecules that regulate autophagy independently of mTOR status, as reviewed in Ref. (109). Thus, mTOR activation and autophagy induction are likely not mutually exclusive processes in all contexts.

#### Decreased Autophagy can Favor Senescence

At variance with the above studies, it has also been reported that inhibition of autophagy promotes senescence (87, 110, 111). Autophagy was reported to counteract senescence by mediating the selective degradation of the transcription factor GATA binding protein 4 (GATA4), which is linked to acquisition of a senescent phenotype in response to DNA damage (112). GATA4 degradation depends on GATA4 recognition by the autophagy receptor p62. Following DNA damage, the p62/GATA4 interaction is reduced, leading to GATA4 stabilization and activation (112). Interestingly, GATA4 activation depends on the DNA damage response regulators, ataxia telangiectasia mutated (ATM), and ataxia telangiectasia and Rad3-related protein (ATR), but not on the traditional senescence effector molecules, p53, and p16 (112). GATA4 may therefore function in DNA damage-induced senescence rather than being a universal senescence-effector molecule.

In addition, a study by Wang et al. adds complexity to the role of autophagy during OIS, as it was reported that genetic ablation of autophagy was permissive rather than restrictive for senescence acquisition during oncogenic RAS-induced senescence (113). In this system, overexpression of Atg5 but not of an autophagy-deficient Atg5 point mutant promoted senescence by-pass, while depletion of Atg5 or Atg3 was permissive for senescence acquisition (113). Induction of OIS was regulated by apoptosis-stimulating of p53 protein 2 (ASPP2) that promoted senescence and inhibited oncogene-induced autophagy through direct disruption of the Atg16–Atg5–Atg12 complex (113), the assembly of which is required for autophagosome formation (114). This suggests a role for ASPP2 in modulating autophagy levels to control the cellular response to oncogene activation. Whether ASPP2 functions in senescence regulation in response to other stimuli remains to be determined. Of note, the ability of autophagy to inhibit OIS appeared not to involve protection from reactive oxygen species or abrogation of p53-activation (113). Understanding the mechanism by which autophagy counteracts senescence in this system may hold the key to combine the contradictory findings on the impact of autophagy on OIS.

Autophagy may also counteract senescence in the context of aging-related senescence and stem-cell maintenance. A study focusing on the regenerative capacity of muscle stem cells using physiologically aged mice, demonstrated that quiescent muscle stem cells preserve their integrity over time through active maintenance of organelle and protein homeostasis by continuous basal autophagy (110). The physiological decline of autophagy in old satellite cells or its genetic impairment in young cells, resulted in accumulation of toxic cellular waste and entry into senescence (110). Similarly, Kang et al. reported that depletion of essential autophagy components resulted in senescence due to build-up of toxic material in primary human fibroblasts (111). The latter studies represent a markedly different experimental system than stress-induced senescence, as they are devoid of external stimuli. Thus, while long-term autophagy inhibition may cause senescence due to accumulation of toxic constituents, autophagy may also function in acute responses to facilitate cellular remodeling in senescence transition in response to conditions such as oncogenic stress or DNA damage.

## Cell Division and Autophagy

Apart from the complex interplay between autophagy and cell-cycle arrest pathways, several studies have reported more specialized regulatory functions for autophagy or autophagy-related factors in the cell division process. Correct segregation of the duplicated genome during cell division is a prerequisite for preventing CIN and aneuploidy, well-described contributors to cellular transformation (115, 116). Involvement of autophagy factors in regulating the progression or fidelity of cell division may thus be an additional component to consider when discussing the intricate relationship between autophagy and tumorigenesis.

### Autophagy and Cytokinesis

In accordance with studies reporting decreased autophagy during mitosis (28, 29), autophagy proteins have primarily been linked to the final phase of cell division, cytokinesis. Cytokinesis is the process in which the two daughter cells are physically separated following chromosome segregation. This is achieved by the formation of a contractile actomyosin ring that constricts the cytoplasm between the segregated reforming nuclei, thereby generating a narrow intercellular bridge. In the center of the bridge is a dense proteinaceous structure termed the midbody ring (MR), which is thought to function as a targeting platform for cleavage factors. Cytokinesis is completed by plasma membrane fission at the intercellular bridge in a process called abscission (117).

A number of studies have reported cytokinesis failure following knock-down of members of the Vps34 complex including Vps34, Beclin 1, Vps15, Bax-interacting factor 1 (BIF-1), and UV irradiation resistance-associated gene (UVRAG) (118–120). The role of the Vps34 complex in cytokinesis regulation is distinct from its function in autophagy induction as it depends on Vps34-mediated production of PtdIns3P at the MR, which functions as a recruitment signal for the FYVE domain-containing cytokinesis regulator FYVE-CENT (120, 121). Accordingly, PI3Kinase inhibition by 3-methyladenine, but not inhibition of autophagy by the lysosome inhibitor bafilomycin A1 or Atg14 depletion, results in abscission failure (120).

The initiation of cytokinesis and mitotic exit is signaled by the anaphase-promoting complex/cyclosome (APC/C) that promotes proteasomal degradation of mitotic regulators including cyclin B; this, in turn, results in CDK1 inactivation and dephosphorylation of its substrates by counteracting phosphatases (122). Vps34 may be one of such CDK1 substrates that are re-activated during the late stages of mitosis after initially being inhibited (29), to participate in the regulation of mitotic exit, although the timing of Vps34 re-activation is not known. Cytokinesis failure can result in the generation of tetraploid cells with supernumerary centrosomes (123). Such tetraploid cells display CIN due to chromosome segregation defects in subsequent cell divisions and are suggested to exhibit increased tumorigenic potential (123–125). Interestingly, the Vps34 complex members Beclin 1, BIF-1, and UVRAG are amongst the autophagy-related proteins with the most well-substantiated tumor suppressor properties (11–14, 16). A detailed dissection of how the individual roles of the Vps34 complex in regulating cytokinesis and autophagy as well as growth factor receptor degradation (126) each contribute to the tumor suppressor function of these proteins, is an important issue for further investigation.

At variance with the studies discussed above, Belaid et al. reported abscission failure upon depletion of Atg5 and in cells derived from lysosomal vacuolar-type H^+^-ATPase *a3*-null mice (127), indicating a function for autophagy in cytokinesis. The cytokinesis defects observed in these systems were attributed to defective turn-over of active RhoA (127), a member of the Rho GTPase family that orchestrates cytokinesis through its ability to regulate the actomyosin contractile network at the cleavage zone (128). Depletion of Atg5 resulted in RhoA enrichment at the intercellular bridge leading to approximately three times broader RhoA activity zones. Consequently, Atg5-depleted cells progressing through mitosis exhibited loose and unstable cleavage furrows and increased generation of multinucleated cells (127). RhoA activity depends on GDP–GTP exchange factors (GEFs) including Ect2, which localizes at the mitotic midbody zone to mediate local RhoA activation and cleavage furrow formation (128, 129). Furthermore, an alternative function for cyclin A2 in potentiating RhoA GTP loading by its GEFs has also been described (130). While the majority of cyclin A2 is degraded by the proteasome in prometaphase (131–133), a small fraction of cyclin A2 was shown to persist in foci later in mitosis and appeared to be subjected to autophagic degradation (40). It is therefore possible that autophagy may have a composite function in controlling appropriate RhoA protein levels and activity at the cytokinesis midzone, by mediating RhoA and Cyclin A2 degradation in late mitosis.

The apparent discrepancies between the reported Vps34 and autophagy-mediated cytokinesis regulation may be most efficiently addressed by expanding these studies to a wider panel of cell systems and autophagy-related proteins. Understanding the contribution of these pathways to cytokinesis completion also *in vivo* is vital for evaluating the potential relevance of these mechanisms in the context of autophagy-related tumor suppression.

In addition, an autophagy-independent function for unc-51-like autophagy-activating kinase 3 (ULK3) as an abscission regulator has been reported (134). Abscission is mediated by the endosomal sorting complexes required for transport (ESCRT) machinery, which mediates membrane remodeling in a number of processes including cytokinesis, viral budding, and autophagy (135). The timing of abscission is regulated by the abscission checkpoint, which delays abscission in response to a number of mitotic abnormalities (136). Interestingly, ULK3 was shown to function in the abscission checkpoint to delay abscission by phosphorylating and binding ESCRT-III subunits in response to lagging chromosomes, nuclear pore defects, and tension forces at the midbody (134). Thus, ULK3 appears to function as an integral part of the abscission checkpoint machinery.

### Autophagy and Cell Division Cleanup

In accordance with the more traditional function for autophagy in cellular maintenance, autophagy may also have a role in returning the cell to its interphase state by clearing leftover structures from normal and abnormal cell divisions.

#### Removal of the MR

Following cytokinesis, the MR is inherited asymmetrically by one of the two daughter cells, and is hereafter often referred to as a MR derivative (MR^d^). MR^d^s can be eliminated by extrusion to the extracellular space (137–139) or by p62/NBR1-mediated selective autophagy (140–142). The NBR1-dependent pathway relies on the interaction between NBR1 and the midbody protein centrosomal protein 55 (CEP55) (141), while the mechanism of p62-mediated MR^d^ degradation and the varying requirement for the two autophagy receptors is not understood. Intriguingly, the MR^d^ extrusion pathway may also involve CEP55 recognition (138). Which elimination pathway predominates varies between cell lines (138), but how MR^d^s are allocated for extrusion or retention and subsequent autophagic degradation is not known. Midbody extrusion likely leads to disposal of both the cytoplasmic and membraneous midbody components, which is not necessarily the case for autophagic degradation; thus, there could be a functional difference between the two midbody disposal pathways. Accumulation of MR^d^s preferentially occurs in stem cells and cancer cells and was suggested to contribute to an undifferentiated phenotype (138, 141). Cells accumulating MR^d^s show decreased autophagic activity and an ability to evade MR^d^ encapsulation and autophagic degradation (141), suggesting a link between autophagy status and MR^d^ accumulation. Nonetheless, MR^d^s remain poorly described structures. How they influence cellular differentiation and their potential tumorigenic relevance is an interesting open question.

#### Removal of Micronuclei

If a cell fails to incorporate all chromosomes and chromosome fragments in the reforming nuclei during cell division, micronuclei can be generated (143). Two studies have observed micronuclei associated with LC3 and LAMP2-stained structures (144, 145), and also colocalizing with charged multivesicular body protein 4B (CHMP4B) (145), a member of the ESCRT machinery. Rello-Varona et al. treated U2OS cells with various cell-cycle inhibitors to increase formation of micronuclei, 2–5% of which colocalized with LC3 and p62, and partially with the lysosome marker LAMP2 (144). Importantly, LC3 colocalization was abrogated upon depletion of Atg5 and Atg7, and electron microscopy further confirmed the presence of micronuclei sequestered within double-membrane vesicles (autophagosomes). LC3-positive micronuclei contained less dense chromatin and discontinuous Lamin B1-stained nuclear envelopes (144), suggesting ongoing digestion. How the autophagy machinery is recruited to micronuclei is, however, not known. Furthermore, as only a small fraction of micronuclei appears to be targeted by autophagy, it remains to be investigated to what extent autophagy contributes to their elimination in comparison to other mechanisms of micronuclei removal, such as extrusion (143).

Of note, the formation of extranuclear chromatin entities does not strictly occur as a result of abnormal mitosis (143). Indeed, Ivanov et al. observed the formation of what was referred to as cytoplasmic chromatin fragments (CCFs) in senescent cells (94). CCFs, in contrast to micronuclei generated from malfunctioning mitosis, were negative for the nuclear lamin A/C and positive for the DNA damage marker γ-Histone 2AX and were generated by nuclear blebbing. CCFs were suggested to be identical to the Lamin B1-associated LADs that were later identified in senescent cells by Dou et al. (89), and intriguingly, both are degraded by autophagy (89, 94). These studies suggest a more general role for autophagy in disposing of extranuclear chromatin.

### Autophagy in Mitotic Arrest and Mitotic Life/Death Decisions

Upon starvation, eukaryotic cells usually arrest in G1 (22). Nonetheless, it has been reported that nitrogen starved budding yeast, lacking essential autophagy genes arrest at the G2/M transition or in mitosis (146, 147). Matsui et al. reported that also nitrogen-starved wild type yeast exhibits a transient G2/M arrest (147). Recovery and progression from this arrest for subsequent G1 block requires autophagy-dependent supplementation of selected amino acids required for cell growth (147). Following replenishment with a nitrogen source, the previously arrested autophagy-deficient cells showed abnormal mitosis associated with a higher incidence of aneuploidy (147). This suggests a role for autophagy in maintaining genome stability by securing arrest in G1 during starvation, at least in budding yeast. Surprisingly, budding yeast may also require autophagy for completing cytokinesis and mitotic exit during nitrogen starvation (146, 147), even though the importance of amino acid supplementation in this context and the relevance of this phenotype in relation to the described mammalian autophagy-related cytokinesis regulation is not fully understood.

In mammalian cells, autophagy may have an important role in determining cell survival during mitotic arrest and mitotic catastrophe. Mitotic catastrophe is a complex oncosuppressive mechanism that is thought to sense mitotic failure and respond by driving cells toward an irreversible fate, be it apoptosis, necrosis, or senescence (148). Autophagy has been shown to facilitate cell survival during mitotic catastrophe (149, 150). Interestingly, during DNA damage-activated mitotic arrest, the previously identified mitosis-related CDK1-mediated phosphorylation of Vps34 on Thr159 (118) promotes Vps34 ubiquitination and proteasomal degradation (80). Degradation is mediated by the p53-responsive gene FBXL20 and the associated Skp1-Cullin-1 complex, and leads to inhibition of autophagy and receptor endocytosis (80). Thus, mitotic Vps34 phosphorylation in the context of p53 activation appears to promote Vps34 degradation (80). Such a mechanism may prevent survival of defective mitotic cells in a dual fashion, by potentially impeding both cytokinesis completion (118) as well as autophagy-dependent cell survival during mitotic arrest.

An alternative function for the autophagy-related protein Atg5 in mitotic catastrophe has also been demonstrated (150). Atg5 was found to be both necessary and sufficient for induction of mitotic catastrophe resulting from sublethal concentrations of DNA-damaging drugs (150). Following these insults, Atg5 translocates to the nucleus, where it physically interacts with survivin and causes the displacement of elements of the chromosomal passenger complex during mitosis, thus resulting in chromosome misalignment and segregation defects, representative of mitotic catastrophe (150). Atg5-mediated mitotic catastrophe does not depend on Atg5–Atg12 conjugation and is unaffected by pharmacological inhibition of autophagy (150); thus, Atg5-mediated mitotic catastrophe occurs independently of its role in autophagy regulation. While the applied drug concentrations only resulted in modest cell death, pharmacological inhibition of the autophagy pathway shifted the response to early caspase-dependent cell death (150), suggesting that in response to DNA damage, cytoplasmic Atg5 and nuclear Atg5 have distinct roles in autophagy induction and mitotic catastrophe, respectively.

Autophagy may under some conditions also participate in promoting mitotic cell death. Doménech et al. reported that, during mitotic arrest caused by abrogation of cyclin B1 degradation, autophagy promotes cell death through ongoing mitophagy (38). The gradual decline in the mitochondrial mass and oxidative respiration, however, resulted in a metabolic switch through activation of AMPK and subsequent induction of glycolysis in a 6-phosphofructo-2-kinase/fructose-2,6-biphosphatase3-(PFKFB3)-dependent manner. Inhibition of glycolysis in breast cancer cells resulted in accelerated death of mitotic cells caused by microtubule poisons (38). This system represents a nonstress-induced mitotic arrest and is likely devoid of p53 activation. Thus, while autophagy induction occurred both during prolonged mitotic arrest (38) as well as in response to DNA damage-induced mitotic catastrophe (150), the resulting effect on cell survival may depend on the nature and severity of the stimulus leading to mitotic block. Of note, exploring how metabolic pathways influence life and death decisions of mitotically arrested cells is of particular interest in the context of cancer treatments, such as DNA-damaging agents or microtubule poisons, which affect the progression and fidelity of mitosis.

### Nutrient Sensing and Cell Division: Involvement of AMPK in Mitosis Regulation

Surprisingly, in recent years, an unexpected mitotic role for the nutrient sensing and autophagy-inducing factor, AMPK, has been discovered. AMPK depletion results in mitotic abnormalities, including spindle misorientation and cytokinesis failure in *Drosophila Melanogaster* S2 cells and human cell lines (151–153). Furthermore, *Drosophila* AMPK-null embryos display severe abnormalities in cell polarity and mitosis (154). AMPK activation, evaluated by AMPK Thr172 phosphorylation (p-AMPK^Thr172^), correlates with induction of mitosis (151, 153), during which p-AMPK^Thr172^ is enriched specifically at centrosomes and at the cleavage furrow (153, 155). Furthermore, an elegant chemical genetics screen designed to identify novel substrates of AMPKα2 provided additional emphasis to a mitotic function for AMPK as it revealed 28 previously unidentified putative AMPK substrates enriched for proteins involved in chromosomal segregation, mitosis, cytokinesis, and cytoskeletal reorganization (151). These evidence indicate a role for AMPK in regulating mitosis through phosphorylation of mitosis-specific substrates. Nonetheless, there appears to be a considerable overlap between the pathways governing AMPK induction and responses during mitosis and during nutrient stress.

Several reports have implicated myosin regulatory light chain (MRLC) as a key target of AMPK-mediated mitosis regulation (151, 153, 154). AMPK facilitates the phosphorylation of MRLC at Serine 19 (151, 154), a phosphorylation event known to stimulate the Mg^2+^-ATPase activity of myosin II leading to actin-based regulation of mitosis, cell migration, and cell polarity (156–159). Accordingly, AMPK depletion decreases the level of p-MRLC^Ser19^ at spindle poles and reduces overall mitotic p-MRLC^Ser19^ levels. MRLC has been suggested to be a direct target of AMPK in *Drosophila* (154), but mammalian cells may employ alternative strategies for AMPK-mediated p-MRLC^Ser19^ regulation. Protein phosphatase 1 regulatory subunit 12C (PPP1R12C) and p21-activated protein kinase (PAK2), both regulators of MRLC phosphorylation status (160–163), were identified as direct targets of AMPK (151). AMPK phosphorylation of these substrates indirectly induces MRLC Ser19 phosphorylation (151). Of note, the AMPK substrate and upstream autophagy regulator ULK1 has also been implicated in the regulation of MRLC phosphorylation (164). Thus, AMPK-induced MRLC phosphorylation may also involve autophagy factors. While Banko et al. identified a number of well-known mitotic regulators as putative AMPK substrates (151), MRLC regulation appears to be a major contributing factor, as depletion of MRLC partially reproduces AMPK depletion phenotypes (153). Moreover, the expression of a phosphomimetic mutant of MRLC is able to rescue AMPK-null-related cell polarity and mitosis defects in *Drosophila* (154). Whether AMPK regulates other substrates during mitosis remains to be determined.

Perhaps, the most intriguing questions in this context remains whether AMPK activation during mitosis is coordinated with its nutrient sensing ability, and if not, which mitosis-specific signals facilitate AMPK activation. Starvation or stress-induced AMPK activation involves allosteric activation by AMP and phosphorylation by upstream kinases on Thr172 in the activation loop of the catalytic α subunit (165). In mammals, the primary kinases performing this task are LKB1 (166–168) and calcium/calmodulin-dependent protein kinase kinase (CAMKK) (169, 170). LKB1 deficiency reproduces the mitotic abnormalities of AMPK deficiency (152, 153, 171), although CAMKK can also promote mitotic AMPK activation in LKB1-deficient systems (153). Thus, mitotic AMPK activation appears to rely on mechanisms resembling those governing starvation-induced AMPK activation. Interestingly, phosphorylation of PPP1R12C, PAK2, and MRLC also occurs in response to energy deprivation (151, 154, 164), indicating that regulation of these factors may be a general response to AMPK-activating stimuli rather than mitosis-specific. Intriguingly, myosin II activation, as indicated by MRLC phosphorylation, is reported to participate in autophagy induction by modulating Atg9 trafficking during starvation (164). Whether autophagy is induced in response to mitotic AMPK activation remains to be investigated.

It is entirely possible that AMPK regulation of mitosis represents a novel function that is unaffected by the cellular energy status, potentially involving selective AMPK activation at specific subcellular localizations during cell division. However, it has also been speculated that AMPK could alternatively promote the completion of already initiated cell cycles in response to energy deprivation to secure proper cell-cycle arrest in the ensuing G1 phase (165). This theory would imply a role for AMPK in initiating responses similar to those reported in yeast, in which autophagy supplies amino acids required for mitotic completion during starvation (147). Thus, intriguing questions for further investigation include understanding the exact mechanism governing mitotic AMPK activation and the requirement for AMPK (and possibly autophagy) for mitotic progression in response to diverse nutrient conditions.

## Concluding Remarks

Autophagy, being traditionally viewed as a bulk process, was initially rarely linked to strictly structured processes, such as cell-cycle progression. Recent advances in the field, however, clearly suggest a strong correlation between autophagy activation and the induction and possibly execution of cell-cycle arrest programs, as well as autophagy (factor) regulation of the cell division process. Cell-cycle stress responses and resulting senescence acquisition constitute important anticancer barriers. Therefore, the relevance of autophagy in executing these responses and the role of autophagy in determining cellular life and death decisions in these contexts are of discernible interest. The role of autophagy and autophagy-related factors in regulating the fidelity of cell division is also potentially of substantial relevance, as findings on this topic suggest that the genomic instability observed upon ablation of autophagy (or specific autophagy components) may be partially attributed to dysregulation of this process. Furthermore, as an increasing number of autophagy proteins are being demonstrated to mediate alternative nonautophagic functions (i.e., PtdIns3K components, Atg5, Atg7, AMPK, AMBRA1, ULK1), we may need to more frequently consider autophagy factors individually. Of note, most evidence linking autophagy and cell-cycle regulation has been obtained in yeast and mammalian cell culture systems and remains to be tested *in vivo*. Thus, an important topic for future investigation includes evaluating the contribution of cell-cycle arrest programs and mitosis regulation to tumor progression or prevention in autophagy-manipulated animal models. While considering cell-cycle (dys)regulation as a factor surely adds to the complexity, it may also open up new avenues for improving our understanding of the intricate relationship between autophagy and tumorigenesis.

## Author Contributions

SM wrote the manuscript and drafted the figures. FC and DZ provided senior supervision and critically revised the manuscript.

## Conflict of Interest Statement

The authors declare that the research was conducted in the absence of any commercial or financial relationships that could be construed as a potential conflict of interest.

## Abbreviations

γH2AX, γ-Histone 2AX; 3-MA, 3-methyladenine; AMBRA1, activating molecule in Beclin 1-regulated autophagy; AMPK, AMP-activated protein kinase; APC/C, anaphase-promoting complex/cyclosome; ASPP2, apoptosis-stimulating of p53 protein 2; Atg 3, 5, 7, 8, 9, 12, 14, autophagy-related gene 3, 5, 7, 8, 9, 12, 14; ATM, ataxia telangiectasia mutated; ATR, ataxia telangiectasia and Rad3-related protein; Bcl-2, B-cell lymphoma 2; Bcl-X_L_, B-cell lymphoma extra large; BIF-1, Bax-interacting factor 1; BNIP3, BCL2-interacting protein 3; CAMKK, calcium/calmodulin-dependent protein kinase kinase; C-Cbl, Casitas B-lineage lymphoma; CCFs, cytoplasmic chromatin fragments; CDK 1, 2, 4, cyclin-dependent kinase 1, 2, 4; CDKIs, cyclin-dependent kinase inhibitors; CDKs, cyclin-dependent kinases; CEP55, centrosomal protein 55; CHMP4B, charged multivesicular body protein 4B; CIN, chromosomal instability; Cip/Kip, CDK interacting protein/kinase inhibitory protein; c-Myc, myelocytomatosis oncogene cellular homolog; DAPK1, death-associated protein kinase 1; Ddit4/Redd1, DNA damage-inducible transcript 4; DRAM, damage-regulated autophagy modulator; Drp1, dynamin-like protein; E2F, E2 factor; Ect2, epithelial cell-transforming sequence 2; ESCRT, endosomal sorting complex required for transport; FBXL20, F-box/LRR-repeat protein 20; GATA4, GATA binding protein 4; GEFs, GDP–GTP exchange factors; GFP, green fluorescence protein; INK4, inhibitors of CDK4; LADs, lamin-associated domains; LAMP2, lysosomal-associated membrane protein 2; LC3, light chain 3; LIR, LC3-interacting region; LKB1, liver kinase B1; MAP1LC3B, microtubule-associated protein 1 light chain 3 beta; Mdm2, mouse double minute 2 homolog; MEFs, murine embryonic fibroblast; MR, midbody ring; MR^d^, midbody ring derivative; MRLC, myosin regulatory light chain; mTOR, mammalian target of rapamycin; NBR1, neighbor of BRCA1 gene; NDP52, nuclear dot protein 52 kDa; OIS, oncogene-induced senescence; PAK2, p21-activated protein kinase; PE, phosphatidylethanolamine; PFKFB3, 6-phosphofructo-2-kinase/fructose-2,6-biphosphatase3; PP2A, protein phosphatase 2 A; PPP1R12C, protein phosphatase 1 regulatory subunit 12C; PRKAB1, 2, protein kinase AMP-activated noncatalytic subunit beta 1, 2; PtdIns3K, class III phosphatidylinositol 3-kinase; PtdIns3P, phosphatidylinositol 3-phosphate; PTEN, phosphatase and tensin homolog; RAS, rat sarcoma; Rb, retinoblastoma protein; RB1CC1/FIP200, RB1 inducible coiled-coil 1/FAK family kinase-interacting protein of 200 kDa; RhoA, Ras homolog family member A; SASP, senescence-associated secretion phenotype; Skp1, S-phase kinase-associated protein 1; smARF, small mitochondrial ARF; SQSTM1, sequestosome 1; TASCC, mTOR-autophagy spatial coupling compartment; TIS, therapy-induced senescence; TSC2, tuberous sclerosis 2; ULK1, 2, 3, unc-51-like autophagy-activating kinase 1, 2, 3; USP 10, 13, ubiquitin-specific peptidase 10, 13; V-ATPase, vacuolar-type H^+^-ATPase; UVRAG, UV irradiation resistance-associated gene; Vps 15, 34, vacuolar protein sorting 15, 34.
